# Learning from tuberculosis: COVID-19 highlights the need for more robust infection control policy

**DOI:** 10.7189/jogh.10.020328

**Published:** 2020-12

**Authors:** Alexander Moran, Matsie Mphahlele, Lindiwe Mvusi, Cindy Dlamini, Sevim Ahmedov, Hala Jassim AlMossawi, Neeraj Kak

**Affiliations:** 1 University Research Co., LLC (URC), Center for Innovation and Technology, Chevy Chase, Maryland, USA; 2 University Research Co., LLC (URC), USAID Tuberculosis South Africa Project, Pretoria, Gauteng, South Africa; 3 National Department of Health, Pretoria, Gauteng, South Africa; 4 United States Agency for International Development (USAID), Pretoria, Gauteng, South Africa; 5 United States Agency for International Development (USAID), Washington, D. C., USA

Coronavirus disease 2019 (COVID-19) represents an unprecedented challenge in modern public health practice. Having spread to over 180 countries and having affected millions of individuals, the COVID-19 pandemic requires a coordinated, effective response without sacrifice to quality or availability of other essential medical services [1]. This rapidly-moving pandemic has laid bare the importance of effective surveillance, quarantine, testing and diagnosis, contact tracing and hospital infection prevention and control measures. Certain countries, including Singapore, have effectively contained community spread of the virus through early and broad quarantine, testing and contact tracing measures [2-4]. In countries like Italy and Spain – where responses were slower, narrower in focus and less consistently implemented – COVID-19 transmission has spread widely in the community, and responses have shifted from containment to mitigation [4,5]. In these countries where containment measures have failed, health systems must plan to ration life-saving medical equipment like mechanical ventilators [6-8]. As COVID-19 spreads to more countries daily, governments begin to prepare for community spread and start to impose travel restrictions, quarantines and physical distancing measures in an effort to “flatten the curve” and to minimize health system strain [9,10].

Minimizing nosocomial (hospital-based) infections is integral to an effective COVID-19 response. The World Health Organization (WHO) has published interim COVID-19 infection prevention and control (IPC) guidance, which focuses on administrative controls (including policy, guidance, training and triage practices), environmental controls (including ventilation and waste management) and personal protective equipment (PPE, including rational use thereof) [11]. As more patients become sicker and require hospitalization, public health leaders must look toward existing hospital-based IPC programs to ensure a broad, consistent prevention and control effort. Tuberculosis (TB) IPC programs are uniquely prepared for this challenge and can be leveraged to mobilize already-trained health care workers, to adapt existing TB IPC guidance documents and to implement existing administrative controls, environmental controls and PPE practices.

Aside from responding to this global emergency, countries must also prepare for future outbreaks and continue to support and strengthen existing infectious disease programs [12]. In this viewpoint, we discuss the readiness of World Health Organization (WHO) Africa Region countries for COVID-19 IPC implementation, offer an example of a successful TB IPC intervention in South Africa, and describe how this intervention can be adapted for the COVID-19 response. We also discuss the need for continued momentum and sustainability for IPC programs before, during and after a pandemic threat.

## COVID-19 INFECTION PREVENTION AND CONTROL READINESS

WHO recently published interim IPC guidance for COVID-19 describes standard airborne IPC practices for hospital settings, which mirror the general WHO minimum requirements for IPC programs [11,13]. The interim guidance includes the following components, with example interventions described within each component:

**Triage and administrative controls**
- Establish a well-equipped triage station at the entrance of health facilities which is supported by trained staff
- Use screening questionnaires according to the most updated case definition
- Post signs in public areas asking symptomatic patients to alert health care workers (HCWs)
- Ensure social distancing (ie, a minimum of 1m between people) or screening people as they enter
- Ensure that patients cover their nose and mouth with a tissue or elbow, scarf or home-made mask
- Offer a medical mask to patients with suspected COVID-19 in waiting rooms or in cohorting areas
- Encourage good hand hygiene with handwashing with soap and water or alcohol gel
- Provide adequate training to HCWs with weekly in-service training
- Establish a surveillance process for possible COVID-19 infection among HCWs
- Monitor HCW compliance with standard IPC precautions
- Restrict visiting hours to facilities as well as the number of people that can visit health facilities
- Ensure posters, patient education and handwashing/sanitizing.

**Environmental and engineering controls**
- Ensure adequate ventilation in rooms where aerosol-generating procedures are performed
- Limit room capacity to the absolute minimum for patient care and support

**Figure:**
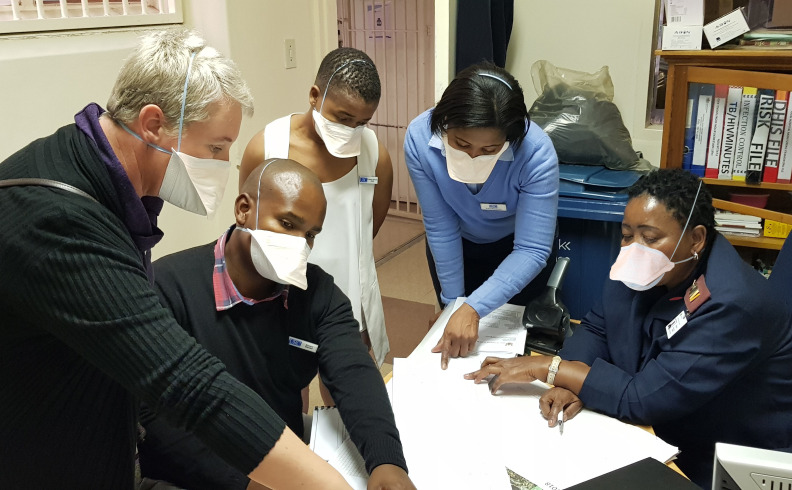
Photo: USAID Tuberculosis South Africa Project staff and the area manager for the TB Ward at Pretoria West Hospital conduct a chart audit as part of the FAST intervention (from the collection of University Research Co., LLC., used with permission).

**Personal Protective Equipment**
- Implement standard PPE precautions
- Implement additional contact and droplet precautions before entering the room of a patient with suspected or confirmed COVID-19
- Use particulate respirators (at least as protective as N95 or FFP2)

In the context of these interim guidelines, WHO published a dashboard summarizing the readiness of countries in the WHO African Region for COVID-19 response [14]. These questions aim to describe IPC readiness and are presented in **Table 1**. We have separated the questions into the three IPC domains: administrative, environmental and PPE. Most questions (n = 7) reflected readiness for implementing administrative controls, and the remaining three questions describe readiness to implement PPE (n = 2) and environmental controls (n = 1). Countries were overall least prepared for administrative control implementation, with an average 58% of countries (n = 48) responding “yes” to administrative control readiness questions. Within administrative controls, countries were overall least prepared for the following questions: “Is there an IPC team responsible to follow up on exposed HCWs? What policies are in place for confirmed cases of HCWs in contact with patients?” (n = 24 responded yes, 51%) and “Is there a triaging system that can be applied to ARI [Acute Respiratory Infections, upper- or lower- respiratory tract infections which include the common cold, pneumonia and influenza] in the health facilities” (n = 26 responded yes, 54%). Countries were more prepared for environmental controls (n = 48 countries, 77% responded yes to n = 1 question) and PPE measures (n = 44 to n = 48 countries, 67% responded yes to n = 2 questions) [15].

**Table 1:** WHO African Region Readiness Response Sheets for infection prevention and control, with yes/no responses overall and for South Africa

			Country responses			South Africa response
**Question**	**IPC domain**	**Responses**	**Yes**	**No**	**% Yes**
Is there an IPC team responsible to follow up on exposed HCWs? What policies are in place for confirmed cases of HCWs in contact with patients?	Administrative	47	24	23	51%	Yes
Is there a triaging system that can be applied to ARI in the health facilities?	Administrative	48	26	22	54%	No
Are health workers trained on IPC measures for respiratory infections?	Administrative	48	28	20	58%	Yes
Are standard precautions applied for all patients with suspected or confirmed high-threat pathogens?	Administrative	48	28	20	58%	Yes
Are there general IPC guidelines in place at both facility and community levels?	Administrative	48	28	20	58%	Yes
Does the HCFs/Health Districts have existing IPC committees or structures?	Administrative	48	29	19	60%	Yes
Is there a functioning national IPC program in place in country?	Administrative	48	33	15	69%	Yes
Is there a system in place for collection and disposal of contaminated medical waste?	Environmental	48	37	11	77%	Yes
Is PPE available for medical staff for screening? If so what PPE is available and how many and in what quantities?	PPE	44	29	15	66%	Yes
Is PPE available for medical staff for case management? If so what PPE is available and how many and in what quantities (are there PAPRS, N95s, Surgical masks, gowns, gloves, etc.)?	PPE	48	33	15	69%	Yes

IPC – infection prevention and control, PPE – personal protective equipment, HCW – health care worker

In examining readiness for COVID-19 spread, we now review the outbreak in South Africa, currently the largest on the continent [1,2]. South Africa responded “yes” to all readiness questions except one administrative control question: “is there a triaging system that can be applied to ARI in the health facilities?” Even in a country like South Africa which is relatively well-prepared for COVID-19, we must examine any additional resources which can be leveraged for an even more effective response. As the country rapidly prepares for mitigating community spread through actions like a nationwide lockdown and travel restrictions, ensuring a coordinated infection prevention and control response at the facility-level can further improve these gains and can ensure that South Africa leverages all possible resources [16,17].

## SUPPORTING EXISTING SYSTEMS IN SOUTH AFRICA FOR A COORDINATED PANDEMIC RESPONSE

South Africa is uniquely positioned to respond swiftly and broadly to emerging community transmission of COVID-19. With the largest HIV burden in the world and a high TB and MDR-TB burden, IPC practices are well understood and buy-in from providers, health officials and other stakeholders is high. To most effectively prepare for COVID-19 spread in hospital facilities, however, South Africa must look at current innovations within the national TB control program and those of TB technical assistance partners.

According to the most recent WHO Global TB report, South Africa is one of eight countries accounting for two thirds of the global TB burden (3% alone) and is listed as one of the 30 high TB burden countries [18]. TB is the leading infectious cause of death globally, killing over one million people in 2018 alone [18]. The disease is caused by *Mycobacterium tuberculosis*, which spreads when someone with TB expels the bacteria into the air, most commonly through coughing. The national TB control program in South Africa, along with key technical assistance partners like the USAID Tuberculosis South Africa Project, continue to develop and implement innovative TB IPC policies and interventions. One of these interventions is an administrative control measure meant to improve triage practices for patients with suspected TB while reducing time from hospital presentation to TB testing, from testing to diagnosis and from diagnosis to treatment initiation. This strategy, known as *Finding cases Actively, Separating safely and Treating effectively* (FAST), is currently implemented in 84% (n = 86) of hospitals in eleven priority districts in South Africa, including OR Tambo, Sarah Baartman, Nelson Mandela Health Metro, Waterberg, Sekhukhune, Mangaung, Fezile Dabi, Johannesburg Health Metro, Tshwane Health Metro, eThekwini and uMkhanyakude [19].

*Finding cases actively:* The first pillar of the FAST strategy is to identify patients with suspected TB upon entry to a supported hospital. Upon entering a facility, a health care worker greets each patient and identifies those with TB symptoms, including those with an active cough, into an area separate from the general waiting area for patients. This process is conducted regardless of hospital ward and is not restricted to TB clinics in order to find as many patients with TB symptoms as possible, even those who may be at the hospital for an unrelated reason.

*Separating safely:* After identifying symptomatic patients, these patients are separated into a waiting area which is separate from the general waiting area, are given a surgical mask and are educated on cough hygiene. Patients are then asked to produce a sputum sample which is used for molecular testing (generally GeneXpert MTB/RIF assay) for identification of TB and resistance to rifampicin, a common first-line TB treatment. After the sample is given to laboratory technicians, a nurse will further separate patients into a separate waiting area. Laboratory technicians then test the sample immediately, and results are available on the same day, sometimes in as little as four hours (in which the sample result is available in two hours, and the result is delivered within four hours after performing internal quality checks and reporting) [20].

*Treating effectively:* If a sputum sample indicates presence of active TB, a nurse will then explain the test result to the patient and begin anti-TB treatment. If rifampicin resistance is indicated, further drug susceptibility testing (DST) including line probe assay (LPA) and other testing may be indicated. Patients are generally started on treatment on the same day before leaving the facility and are referred to a lower-level health center for local, decentralized follow up care.

The FAST approach has identified 12 636 additional TB cases which would have otherwise been missed. Additionally, the Tuberculosis South Africa Project has achieved high screening, testing and treatment initiation rates among patients entering supported facilities as shown in **Table 2**. Additional facilities have adopted the FAST approach in an incremental fashion, and The Tuberculosis South Africa Project has improved the proportion of patients screened from 37.6% in Q2 of 2017 to 64.8% in Q4 of 2019. Over the same time, the project improved the proportion of patients tested with GeneXpert MTB/RIF (GXP) from 40.8% to 66.5%. Finally, the project has maintained high treatment initiation rates of drug-sensitive TB (DS-TB) (90.2% in Q4 of 2019) and has improved the proportion of patient started on rifampicin-resistant TB (RR-TB) from 37.8% in Q2 of 2017 to 84.8% in Q4 of 2019.

**Table 2:** USAID Tuberculosis South Africa Project FAST Results in supported districts, 2017-2019

Year	Quarter	Patients screened (N)	Patients screened (% of Headcount)	Patients Tested with GeneXpert (N)	Patients tested with GeneXpert (% of presumptive)	Patients diagnosed with DS-TB (N)	Patients diagnosed with DR-TB (N)	DS-TB treatment initiation (% of diagnosed)	DR-TB treatment initiation (% of diagnosed)
2017	Q2	46 328	31.4%	1940	31.0%	238	45	85.3%	37.8%
	Q3	91 649	37.7%	4298	41.5%	601	159	75.5%	8.8%
	Q4	154 183	56.3%	3355	67.0%	642	392	39.9%	3.3%
2018	Q1	171 867	52.8%	3092	61.3%	576	455	22.6%	2.9%
	Q2	241 496	64.3%	6046	65.6%	796	476	41.1%	4.8%
	Q3	436 083	63.0%	13138	71.8%	1352	121	89.4%	52.1%
	Q4	438 674	67.6%	8383	63.2%	1473	143	88.9%	32.2%
2019	Q1	549 300	69.8%	12 042	69.2%	1457	71	92.5%	87.3%
	Q2	512 345	71.9%	10 783	60.7%	1180	54	90.7%	88.9%
	Q3	603 022	61.9%	11 015	70.0%	1452	54	87.1%	70.4%
	Q4	399 464	66.3%	6442	63.4%	851	48	88.2%	87.5%
**Total**		**3 644 411**	**63.0%**	**80** **534**	**62.7%**	**10** **618**	**2018**	**78.4%**	**18.8%**

DS-TB – drug-susceptible tuberculosis, DR-TB – drug-resistant tuberculosis

Aside from the FAST initiative, the Tuberculosis South Africa Project works extensively in governance and policy guidance, contact management, environmental controls and awareness building activities to reduce stigma surrounding TB and to encourage people to get tested and start TB treatment. Taken together, these TB-specific IPC interventions serve as useful entry points for COVID-19 IPC responses. Importantly, these TB interventions are functional, have political buy-in and funding, have trained staff and facility-level champions, and have proven results. Health officials must look to functioning programs like these to mount a wide-ranging, agile response to the public health emergency that is COVID-19.

## CONTINUING THE MOMENTUM: TOWARD A UNIVERSAL CULTURE OF INFECTION PREVENTION AND CONTROL

While South Africa serves as one example, other countries will necessarily be affected by the COVID-19 pandemic. Other studies including ecological analyses should be implemented to understand possible country-level associations between TB and COVID-19 burden, especially among high TB burden countries. As the body of evidence grows, more specific recommendations can be made to more effectively prepare for future airborne infection threats.

COVID-19 represents an unprecedented challenge for infection prevention and control. As shown in settings like South Africa, however, there are successful and functional TB IPC programs in place which can be mobilized quickly and universally to prevent widespread hospital-based infections. In using these strategies, TB program managers and other stakeholders must harness this opportunity for a sustained focus on acute respiratory infections and airborne infection control programs. TB is the biggest infectious disease killer globally and, while COVID-19 is a global public health emergency, we must maintain this urgency and momentum for TB IPC after the threat has passed.

As the world comes together to tackle the COVID-19 pandemic, it is important to ensure that essential services and operations for dealing with long-standing health problems continue to protect the lives of people with TB and other diseases or health conditions. Health services, including national programs to combat TB, need to be actively engaged in ensuring an effective and rapid response to COVID-19 while ensuring that TB services are maintained [21].

In funding and bolstering these existing TB IPC strategies for COVID-19 responses, country governments can improve airborne IPC interventions in general, to improve TB case detection, to reduce nosocomial infections and to reduce TB deaths generally. While the initial benefit in expanding these programs lies in preventing wide community transmission of COVID-19, we must recognize the opportunity in maintaining this level of response against TB to continue to reduce new TB infections and deaths from TB.

## CONCLUSION

As COVID-19 continues to spread globally, we must look to existing health system capacity for a rapid and robust response. As shown by WHO, IPC readiness on the African continent varies and shows only moderate preparedness for impending outbreaks, in which South Africa represents the ideal scenario. In high TB burden countries like South Africa, IPC interventions, especially like those implemented by the USAID Tuberculosis South Africa Project, offer a ready-made entry point to broad airborne IPC responses with trained providers, guidance documents and facility-level champions. These programs must be expanded and resourced to minimize nosocomial infection and death. In expanding these programs, however, we must keep the future in mind and realize the 2-fold benefit of IPC programs: the immediate benefit of preventing COVID-19 outbreaks, and the long-term benefit of reducing ARI death in general, including deaths form TB. In the wake of World TB Day and in the context of a rapidly moving pandemic, there is no better resource for high burden TB countries than national TB control programs. We must rapidly expand and resource these programs to evade unnecessary death and suffering due to COVID-19 and other ARIs.
